# Novel Insights and Features of the NDM-5-Producing Escherichia coli Sequence Type 167 High-Risk Clone

**DOI:** 10.1128/mSphere.00269-20

**Published:** 2020-04-29

**Authors:** Aurora Garcia-Fernandez, Laura Villa, Giulia Bibbolino, Alessia Bressan, Maria Trancassini, Valeria Pietropaolo, Mario Venditti, Guido Antonelli, Alessandra Carattoli

**Affiliations:** a Department of Infectious Diseases, Istituto Superiore di Sanità, Rome, Italy; b Department of Molecular Medicine and Medical Biotechnologies, University of Naples Federico II, Naples, Italy; c Department of Molecular Medicine, Sapienza University of Rome, Rome, Italy; d Department of Public Health and Infectious Diseases, Sapienza University of Rome, Rome, Italy; JMI Laboratories

**Keywords:** *Escherichia coli*, capsular polysaccharide, carbapenems

## Abstract

Escherichia coli sequence type 167 (ST167), producing the metallo beta-lactamase NDM-5, has been isolated as a colonizer of patients recovered at the University Hospital Policlinico Umberto I of Rome. Phylogenesis and comparative analysis of the genomes of these strains were performed against 343 ST167 genomes available from the EnteroBase database. These analyses revealed that resistance plasmids, integrative conjugative elements (ICEs), carrying the yersiniabactin virulence trait and capsular synthesis gene clusters had variable compositions and distributions within different strains of the ST167 clone. A novel capsular synthesis gene cluster, highly similar to the K48 cluster previously described only for Klebsiella pneumoniae, was identified in phylogenetically related strains of the ST167 clone.

**IMPORTANCE** Global dissemination of some E. coli high-risk clones has been described in the last decades. The most widespread was the ST131 clone, associated with extended-spectrum-beta-lactamase (ESBL) production. Genomics of ST131 demonstrated that one clade within the ST emerged in the early 2000s, followed by a rapid, global expansion. The E. coli ST167 clone is emerging throughout the world, being frequently reported for its association with carbapenem resistance. Our study shows that virulence features are differently represented within the ST167 population. One clade shows the K48 capsular synthesis gene cluster of K. pneumoniae, not previously described for E. coli, and is populated by NDM-5-producing strains. The combination of resistance and virulence may sustain the global expansion of this specific ST167 clade.

## Abstract (precis)

Global dissemination of some E. coli high-risk clones has been described in the last decades. The most widespread was the ST131 clone, associated with extended-spectrum-beta-lactamase (ESBL) production. Genomics of ST131 demonstrated that one clade within the ST emerged in the early 2000s, followed by a rapid, global expansion. The E. coli ST167 clone is emerging throughout the world, being frequently reported for its association with carbapenem resistance. Our study shows that virulence features are differently represented within the ST167 population. One clade shows the K48 capsular synthesis gene cluster of K. pneumoniae, not previously described for E. coli, and is populated by NDM-5-producing strains. The combination of resistance and virulence may sustain the global expansion of this specific ST167 clade.

## INTRODUCTION

Health care-acquired infections caused by carbapenem-resistant bacteria considerably affect the mortality of infected patients and impact health care costs. Such infections are recognized as one of the most relevant threats to public health worldwide (1). The New Delhi (NDM) metallo-beta-lactamase (MBL) is able to hydrolyze most beta-lactams, including carbapenems. The highest prevalence of NDM-positive *Enterobacterales* is in the Indian subcontinent, the Middle East, and the Balkans (2). The Italian surveillance of antibiotic resistance showed that from 2009 to 2013, the percentage of carbapenem-resistant Escherichia coli was only 1.9% among carbapenemase-producing *Enterobacterales* (CPE), while the incidence of Klebsiella pneumoniae was reported to be 98.1%. In most of the CPE strains the enzyme reported was KPC (in 95.2% of K. pneumoniae strains), while MBLs were rarely reported. The data stem from passive surveillance of invasive infections that may significantly underestimate the prevalence of MBL-producing E. coli strains colonizing patients or causing urinary tract infections (UTIs) (3–5).

More recently, the epidemiological situation in Tuscany, Italy, dramatically changed. Between November 2018 and October 2019, a large and persistent outbreak occurred, caused by NDM-producing *Enterobacterales*; 1,270 (77.2%) cases of intestinal carriage, 129 (7.8%) bloodstream infections, and 246 (14.9%) infections/colonizations at other sites were reported (6). The large majority of confirmed NDM-positive strains were sequence type 147 (ST147) K. pneumoniae (90.9%), followed by E. coli (4.2%), whose STs and *bla*_NDM_ gene variants were not studied (6).

Rapid identification of colonized patients and screening for CPE at the admission of patients in critical wards of the hospital are the most effective actions for reducing health care-associated infections. Screening of CPE is routinely performed by rectal swabs on patients admitted in some critical units of the University Hospital Policlinico Umberto I in Rome.

From September 2018 to March 2019, NDM-producing E. coli colonized or infected five patients in three different wards of our hospital. The occurrence, in a limited period, of five NDM-positive cases in this hospital was followed by intensification of surveillance and infection control measures. A genomic approach was used to study the phylogenetic relationship among the isolates, with the aim to identify a possible interward outbreak occurring within the hospital.

## RESULTS

### Isolation and bacterial typing of NDM-producing Escherichia coli.

In the period from September 2018 to March 2019, a total of five *bla*_NDM_-positive E. coli strains, identified by Xpert Carba-R (Cepheid, Sunnyvale, CA), were isolated at the microbiology laboratory of the University Hospital Policlinico Umberto I of Rome. The first E. coli NDM producer (strain 91_NDM-5) was cultured from a rectal swab sample from a Bangladeshi patient at the hospital admission in September 2018. In 3 weeks, two additional patients from the same ward were found to be colonized by NDM-producing E. coli (92_NDM-5 and 101_NDM-5). In October 2018, an NDM-producing E. coli strain (100_NDM-5) was isolated from a urine sample from a patient in a different ward of the hospital, and in March 2019 a fifth NDM-producing E. coli strain (0311_NDM-5) was obtained from a rectal swab from a patient in a third ward of the same hospital. The 100_NDM-5 and 0311_NDM-5 cases had no identifiable common links with the three previous cases.

All strains appeared mucoid on the plates and encapsulated, as demonstrated by India ink coloration (data not shown). All strains were ST167 by multilocus sequence typing (MLST) and showed resistance to meropenem, ertapenem, imipenem, amoxicillin-clavulanic acid, third- and/or fourth-generation cephalosporins, ciprofloxacin, and piperacillin-tazobactam but remained susceptible to nitrofurantoin, colistin, and tigecycline. Strains 91_NDM-5, 92_NDM-5, and 101_NDM-5 were also resistant to amikacin.

Whole-genome sequencing (WGS) was performed on strains 91_NDM-5, 92_NDM-5, 100_NDM-5, and 0311_NDM-5. Strain 101_NDM-5 did not resuscitate after storage at –80°C and was not further studied.

### Resistance and plasmid content.

The four sequenced ST167 strains shared a common resistance gene pattern including the *bla*_NDM-5_, *dfrA12*, *sul1*, *tet*(A), *aadA2*, and *mph* genes, but other resistance genes were differently sorted among the strains (Table 1). Resistance to amikacin in strains 91_NDM-5 and 92_NDM-5 correlated with the presence of the 16S RNA methylase *rmtB*, while gentamicin resistance in strains 100_NDM-5 and 0311_NDM-5 was conferred by the *aac(3)-IIa* gene.

**TABLE 1:** Features and characteristics of ST167 Escherichia coli analyzed in this study (BioProject PRJNA545093)

Strain	Serotype	Beta-lactamase(s)				Other resistance genes	IncF [FAB formula]^*a*^	IncI1, IncN [pMLST]^*a*^	Other plasmid(s)	ICE^*b*^
91_NDM-5	O89b:H9	NDM-5		CMY-42	OXA-1, TEM-1B	*dfrA12*, *catA1*, *sul1*, *tet*(A), *aadA1*, *aadA2*, *mphA*, *rmtB*	p91_NDM-5 [F36:A4:B-]	p91_I1_CMY-42 [A5-R4-T15]	ColRNAI, Col8282	Neg
92_NDM-5	O89b:H9	NDM-5		CMY-42	OXA-1, TEM-1B	*dfrA12*, *catA1*, *sul1*, *tet*(A), *aadA1*, *aadA2*, *mphA*, *rmtB*	p92_NDM-5 [F36:A4:B-]	p92_I1_CMY-42 [A5-R4-T15]	ColRNAI, Col8282	Neg
100_NDM-5	O89b:K48:H9	NDM-5	CTX-M-1			*dfrA12*, *sul1*, *tet*(A), *aadA2*, *aac(3)-IIa*, *mphA*	p100_NDM-5 [F36:31:A4:B1]	p100_N_CTX-M-1 [ST1]	ColRNAI	Pos
0311_NDM-5	O89b:K48:H9	NDM-5	CTX-M-32			*dfrA12*, *sul1*, *tet*(A), *aadA2*, *aac(3)-IIa*, *mphA*	p0311_NDM-5 [F36:31:A4:B1]	p0311_I1_CTX-M-32 [ST304]	ColRNAI, IncX1	Pos

a In brackets are allele numbers and sequence types assigned by plasmid pMLST at https://cge.cbs.dtu.dk/services/.

b Neg, negative; Pos, positive.

**FIG 1:**
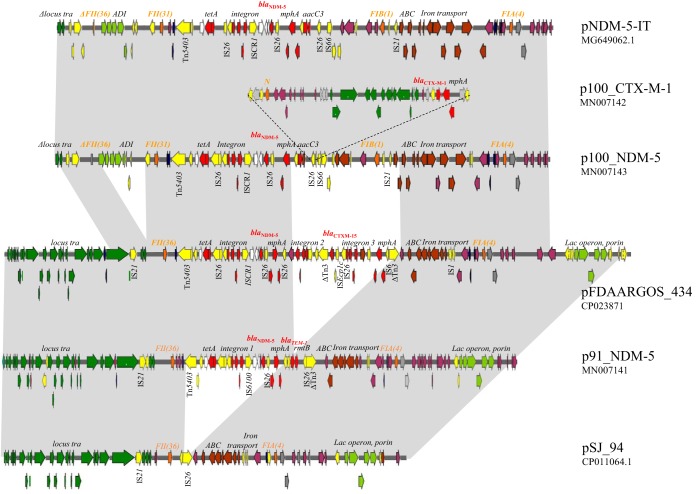
Major structural features of p100_NDM-5 and p92_NDM-5 plasmids in comparison with the reference pSJ_94. Predicted coding sequences are indicated by colored arrows oriented in the direction of transcription of each respective gene: resistance genes, red; transposon-related genes and insertion sequences, yellow; replicons, orange; ABC and iron uptake clusters, brown; ADI and lactose operons, pale green; transfer locus dark green; and toxin-antitoxin genes, blue.

The *bla*_NDM-5_ gene was located on IncF plasmids in all strains; however, different plasmid scaffolds and resistance content were found in strains 91_NDM-5 and 92_NDM-5 than in strains 100-NDM-5 and 0311_NDM-5. Two complete sequences of IncF plasmids carrying *bla*_NDM-5_ were obtained, here named p91_NDM-5 (GenBank accession no. MN007141) and p100_NDM-5 (MN007143), respectively (Fig. 1). p91_NDM-5 identified in strains 91_NDM-5 and 92_NDM-5 was similar to pSJ_94 (CP011064.1), a plasmid identified in 2011 in ST167 strains Sanji from pheasants in China. pSJ_94 did not carry resistance genes, had the FAB formula F36:A4:B-, and carried an iron uptake transport system flanked by an ABC efflux pump gene cluster. p91_NDM-5 had the same structure as pSJ_94 but acquired the *bla*_NDM-5_ gene in a complex integron with the *aadA2* and *dfrA12* resistance gene cassettes, the IS*CR1* element, and a copy of the *intI1* gene with a deletion of IS*26* (Fig. 1).

An IncIγ plasmid carrying the *bla*_CMY-42_ gene (p91_CMY-42, MN007140) was detected in strains 91_NDM-5 and 92_NDM-5. It was characterized by the loss of the *pilV* and *sogS* pMLST genes. The characteristic ardA-5, repI1-4, trbA-15 (A5-R4-T15) pMLST allele combination was identically recognized in several ST167 genomes in GenBank, all positive for the *bla*_CMY-42_ gene (Tables 1 and 2).

**TABLE 2:** Features and characteristics of ST167 Escherichia coli complete genome sequences^*a*^

Strain (accession no.)	Country	Serotype (origin of the capsular locus)	Beta-lactamases	Other resistance genes	IncF [FAB formula] (accession no.)	IncI1 [pMLST] (accession no.)	Other plasmids (accession no.)	Ybt	T4SS
51008369SK1 (CP029973)	Switzerland	O89b:K48:H9 (Klebsiella pneumoniae)	NDM-5, CMY-2, TEM-30	*erm*(B), *aadA1*, *dfrA1*, *floR*, *sul1*, *sul2*, *aac(3)-IIa*, *aadA2*, *dfrA12*, *mph*(A), *sul1*, *tet*(A)	p51008369SK1_E [F36:F31:A4:B1] (CP029978), p51008369SK1_A [F2:A-:B-] (CP029974)	p51008369SK1_B [ST303] (CP029975)	IncX4 (CP029976), IncI2 (CP029977)	Pos	Pos
ECOL-18-VL-LA-PA-Ryan-0026 (CP041392.1)	USA	O89b:K48:H9 (Klebsiella pneumoniae)	NDM-5, CTX-M-15, OXA-1, TEM-1D	*dfrA17*, *dfrA12*, *tet*(A), *mph(A)*, *sul1*, *aadA2*, *aadA5*, *aac(6')Ib-cr*, *aac(3)-IId, aadA22*, *floR*	p45407_1 [F36:A4:B-] (CP041393)	p45407_2 [ST80] (CP041394)		Pos	Pos
FDAARGOS_434 (CP023870)	Canada	O89b:K48:H9 (Klebsiella pneumoniae)	NDM-5, CTX-M-15	*aadA2*, *aadA5*, *dfrA12*, *dfrA17*, *mph*(A), *sul1*, *tet*(A)	Unnamed1 [F36:A4:B-] (CP023871)		Col440_I (CP023868), Col440_I (CP023869), ColND (CP023872)	Pos	Pos
CRE1493 (CP019071)	Hong Kong	O89b:K48:H9 (Klebsiella pneumoniae)	NDM-5, CTX-M-15, OXA-1, TEM-1A	*mcr-1*, *aac(*3*)-IId*, *aadA2*, *dfrA12*, *floR*, *oqxA*, *oqxB*, *strA*, *strB*, *sul2*, *tet*(A), *aac(3)-IIa*, *aac(6')Ib-cr*, *aadA5*, *catB3*, *dfrA17*, *mph*(A), *sul1*	p1493-5 [F36:A4:B-] (CP019076), p1493-3 [F-:A-B68] (CP019074)		IncX3_NDM-5 (CP019073), IncX4 (CP019072), IncY (CP019075)	Pos	Pos
SCEC020007 (CP025627)	China	O89b:K48:H9 (Klebsiella pneumoniae)	NDM-5, TEM-1B	*aadA2*, *aadA5*, *dfrA12*, *dfrA17*, *mph*(A), *rmtB*, *sul1*, *tet*(A)	pNDM5_020007 [F36:A4:B-] (CP025626)		IncB/O/K (CP025625)	Pos	Pos
ECONIH6 (CP026199)	USA	O89b:K48:H9 (Klebsiella pneumoniae)	NDM-5, CTX-M-15, TEM-1B	*dfrA14*, *qnrS1*, *strA*, *strB*, *sul2*, *tet*(A), *aadA2*, *dfrA12*, *erm*(B), *mph*(A), *rmtB*, *sul1*	pNDM-d2e9 [F2:A-:B75] (CP026201)		IncY_CTX-M-15 (CP026200)	Neg	Neg
AR_0011 (CP024855)	ND	O89b:O9:K30:H9 (Klebsiella pneumoniae)	CTX-M-15, OXA-1	*strA*, *strB*, *sul2*	tig00001011_pilon [F36:F31:A4:B1] (CP024856)		IncN-R (CP024857), IncI2 (CP024858)	Pos	Pos
AR_0014 (CP024859)	ND	O89b:O9:K30:H9 (Klebsiella pneumoniae)	CTX-M-15, OXA-1	*aac(3)-IIa*, *aac(6')Ib-cr*, *catB3*, *tet*(A)	unitig_1_pilon[F36:F31:A4:B1] (CP024860)		IncX4 (CP024861)	Pos	Pos
AR_0150 (CP021736)	ND	O89b:K30:H9	NDM-5, CMY-42, TEM-1B	*aadA5*, *dfrA17*, *mph*(A), *sul1*, *tet*(A)	tig00000255 [F36:A4:B-] (CP021737)	tig00002897alt [A4-R3-T15] (CP021739)	IncX3_NDM-5 (CP021738)	Pos	Neg
AR_0151 (CP021691)	ND	O89b:K30:H9	NDM-5, CMY-42			tig00001252 [A4-R3-T15] (CP021693)	IncX3_NDM-5 (CP021692)	Pos	Neg
AR_0149 (CP021532)	ND	O89b:K30:H9	NDM-7, CMY-42			tig00000220 [A5-R4-T15] (CP021533)	IncX3_NDM-7 (CP021534)	Pos	Neg
AR_0162 (CP021683)	ND	O89b:K30:H5	NDM-7, CTX-M-15, TEM-1B	*qnrS1*, *strA*, *strB*, *sul2*, *tet*(A), *erm*(B), *mph*(A)	tig00008015 [F2:A-:B-] (CP021684)		IncX3_NDM-7 (CP021682), IncY_CTX-M-15 (CP021681), tig00002623 (CP021680)	Neg	Neg
Y5 (CP013483)	China	O89b:KL115:H4 (Klebsiella pneumoniae)	CTX-M-14, CTX-M-15, CMY-42, OXA-1	*aac(6')-Ib-cr*, *tet*(A), *dfra17*, *aadA5*, *sul1*, *mph*(A), *floR*, *aph(3')-Ia*	pECY55 [F36:A4: B-] (KU043115.1)	pECY56 [A4-R1-T15] (KU043116)	IncA (KT997783)	Pos	Neg
AR_435 (CP029115)	ND	O89b:K31:H9	NDM-1, CMY-42, OXA-9, TEM-1A, SHV-12	*aadA1*, *aadA2*, *aadA5*, *mph*(A), *sul1*, *tet*(A), *aac(6')-Ib*, *aac(6')Ib-cr, armA*, *dfrA12*, *mph(*E), *msr*(E), *strA*, *strB*, *sul2*	Unnamed1 [F36:A4:B-] (CP029113)	Unnamed6 [A5-R4] (CP029119)	IncC_NDM-1 (CP029118), ND3_CMY-42 (CP029116), ColRNAI (CP029114), ColND (CP029120), ColRNAI (CP029121), ND4 (CP029117)	Neg	Neg
Sanji (CP011061)	China	O89b:K31:H9	CTX-M-14, OXA-1	*aac(*3*)-IVa*, *aac(6')Ib-cr*, *aadA1*, *aadA2*, *aph(3')-Ia*, *aph(*4*)-Ia*, *ARR-3*, *catB3*, *cmlA1*, *floR*, *mph*(A), *oqxA*, *oqxB*, *sul1*, *sul2*, *sul3*, *tet*(M)	pSJ_94 [F36:A4:B-] (CP011064), pSJ_82 [F2:A-:B-] (CP011065)		IncHI2-IncN (CP011062), IncY (CP011063), ColND (CP011066), Col440_I (CP011067)	Neg	Neg
M217 (AP019189)	Myanmar	O89b:K31:H9	NDM-5, TEM-1B	*aadA2*, *dfrA12*, *erm*(B), *mph*(A), *rmtB*, *sul1*	pM217_FII [F2:A-:B-] (AP018147)	pM217_I1 [A5-P3-R4-T15] (AP019190)		Neg	Neg
WCHEC005237 (CP026580)	China	O89b:K31:H9	NDM-5, CTX-M-55, TEM-1B	*dfrA14*, *floR*, *qnrS1*, *tet*(A), *fosA*, *aac(6')Ib-cr*, *aadA16*, *ARR-3*, *dfrA27*, *rmtB*, *sul1*, *tet*(A)	pRmtB1_005237 [F47:A-:B-] (CP026579)		IncX3_NDM-5 (CP026577), IncF (CP026576), IncFIBk (CP026578), ColND (CP026575), ColRNAI CP026574), ColRNAI (CP026573)	Neg	Neg
CREC-629 (CP024815)	South Korea	O89b:K54:H10 (Roultella planticola)	NDM-7, TEM-1B		pCREC-629_1 [F36:F22:A1:B-] (CP024816)		IncX3_NDM-7 (CP024818), IncY (CP024817)	Neg	Neg
CREC-532 (CP024830)	South Korea	O89b:K54:H10 (*Roultella planticola*)	NDM-7, TEM-1B		pCREC-532_1 [F36:F22:A1:B-] (CP024831)		IncX3_NDM-7 (CP024833), IncY (CP024832)	Neg	Neg

a Resistance genes, plasmid incompatibility groups, and replicon alleles were assigned by ResFinder, PlasmidFinder, and *in silico* plasmid multilocus sequence typing (pMLST) at https://cge.cbs.dtu.dk/services/. ND, not determined; Ybt, yersiniabactin; T4SS, type IV secretion system associated with yersiniabactin.

The p100_NDM-5 plasmid identified in strains 100_NDM-5 and 0311_NDM-5 showed 99% nucleotide identity and 99% coverage with pNDM-5-IT described in 2016 for ST167 E. coli strains causing UTIs in a long-term facility in Ancona, Italy. Both plasmids were characterized by the FAB formula F36:F31:A4:B1 and the presence of the arginine deaminase (ADI) virulence factor (7). The ADI cluster carrying the *arcA*, *arcB*, *arcC*, and *arcD* genes and an additional FII31 replicon were acquired together in an IS*66*-IS*1* module flanked by two inverted repeats.

The p100_NDM-5 plasmid showed almost complete deletion of the transfer locus; however, transconjugants were obtained from strain 100_NDM-5 at low efficiency (approximately 1 × 10^−6^ per recipient cell). WGS of one of the transconjugants demonstrated that conjugation was promoted by plasmid fusion with a helper, a self-conjugative plasmid (IncN-IncF fusion; GenBank accession no. MT199177). The helper plasmid, named p100-CTX-M, was of the IncN type and carried the *bla*_CTX-M-1_ gene (MN007142). The fusion of the two plasmids in the transconjugant occurred probably by an IS*26*-mediated recombination event in IS*26* linked to the *aac(3)-IIa* gene (Fig. 1). p100-CTX-M not fused with p100_NDM-5 was demonstrated by WGS of the transformant of strain 100_NDM-5.

### ST167 phylogenesis.

**FIG 2:**
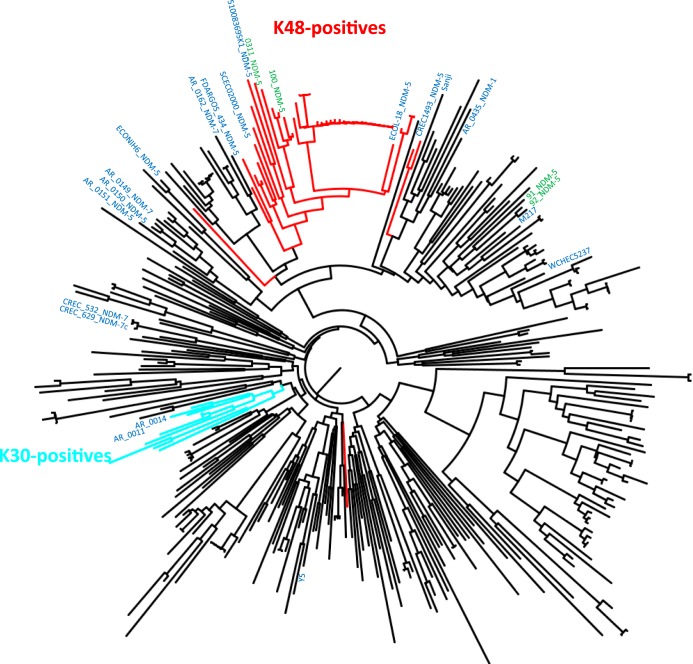
Neighbor-joining phylogenetic tree of ST167 E. coli genomes. The NJ tree was built on an SNP analysis performed on 343 ST167 E. coli genomes, 4 determined in this study and the other downloaded from the EnteroBase database (Table 2). Green-labeled names indicate the four ST167 genomic sequences obtained in this study. The names of complete, circular genomes available in GenBank and used as references for genome synteny are colored in blue. Branches of genomes positive for the K. pneumoniae K48-like capsular cluster are colored in red, and those positive for the K30-like capsular cluster are colored in pale blue.

A total of 343 ST167 genomes available from the EnteroBase database were downloaded and compared with the strains sequenced in this study, generating a single-nucleotide-polymorphism (SNP)-based neighbor-joining (NJ)-phylogenetic tree with multiple clades (Fig. 2). 91_NDM-5 and 92_NDM-5 genomes clustered together (differing from each other by 218 SNPs). 100_NDM-5 and 0311_NDM-5 genomes were each other highly related (404 SNPs) and clustered on a different branch than the 91_NDM-5 and 92_NDM-5 pair. The two pairs of isolates from different wards of the hospital were not related to each other (91_NDM-5 and 100_NDM-5 differed by 3,350 SNPs).

### ST167 genome synteny.

Nineteen complete, circular ST167 genomes were available in the NCBI nucleotide GenBank (indicated by blue names in Fig. 2). These genomes were downloaded and analyzed for genome synteny by SEED Viewer version 2.0 (http://rast.nmpdr.org/seedviewer.cgi).

Among them, six ST167 complete genomes were located in the same branch in the SNP phylogenetic tree as 100_NDM-5 and 0311_NDM-5; they were from Switzerland (51008369SK1 [CP029973]), Canada (FDAARGOS_434 [CP023870]), China (SCEC020007 [CP025627]), the United States (EcoNIH6 [CP026199] and ECOL-18-VL-LA-PA-Ryan-0026 [CP041392.1]), and Hong Kong (CRE1493 [CP019071]), differing by a minimum of 347 SNPs (51008369SK1 and 100_NDM-5) up to 2,967 SNPs (EcoNIH6 and 100_NDM-5).

Genome synteny performed using the 51008369SK1 strain (CP029973) as a reference genome against the 19 complete genomes, and the 4 genomes sequenced in this study, identified 5 major regions of discontinuity. These regions encoded capsular biosynthesis and contained integrative conjugative elements (ICEs) or prophages (Fig. S1 shows one of the results of the synteny studies).

10.1128/mSphere.00269-20.1 FIG S1 Genome synteny among ST167 genomes. The comparison was obtained at the RAST server (http://rast.nmpdr.org/seedviewer.cgi) by BLASTP analysis of the coding sequences, visualized by the Seed Viewer version 2.0. The ST167 coding sequences annotated from genomes listed in the legend were compared with those deduced from the 51008369SK1 (CP029973) complete genome, used as a reference. The major differences among the genomes of the ST167 clone consisted of prophages (ф), ICE, and capsular loci. Download FIG S1, PDF file, 0.1 MB. Copyright © 2020 Garcia-Fernandez et al. 2020 Garcia-Fernandez et al. This content is distributed under the terms of the Creative Commons Attribution 4.0 International license.

**FIG 3:**
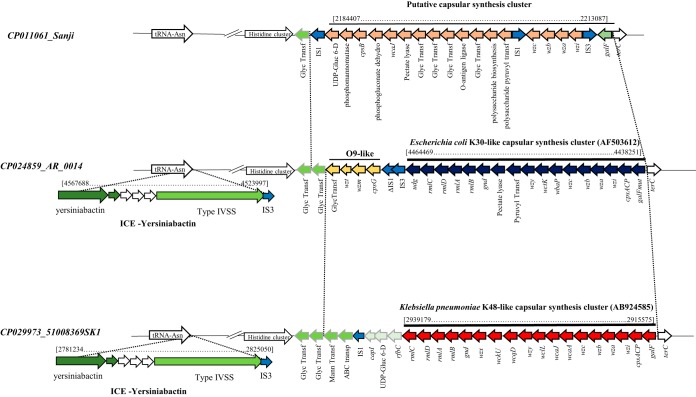
Representation of capsular clusters and integrative conjugative elements (ICEs) identified in ST167 genomes. Arrows indicate genes and their direction of transcription. Three genomes among those studied (listed in Table 2) were selected for drawing representative capsular clusters: Sanji (CP011061), carrying the putative capsular cluster (orange arrows); AR_0014 (CP024859), carrying the E. coli K30 type (AF503612; dark blue arrows); and 51008369SK1 (CP029973), carrying the K. pneumoniae K48 type (AB924585, red arrows). Vertical dotted lines highlight the variable genetic content comprised between two constant regions represented by white arrows. Pale blue arrows indicate insertion sequences and green arrows other genes identified in these variable regions, including a putative O9-like antigen synthesis cluster. Gray arrows indicate ICE-associated genes, encoding the yersiniabactin system, the type IV secretion system, and hypothetical proteins, as identified in the tRNA Asn integration sites. Brackets indicate nucleotide positions of ICEs and capsular clusters in their respective ST167 reference genomes.

A 23,600-bp region of discontinuity was analyzed in 51008369SK1 (Fig. 3). This region revealed an intact capsular synthesis cluster identified by BLASTN similar at 98.8% nucleotide identity and with 99% coverage to the K. pneumoniae K48 capsule (AB924585). This cluster carried the K. pneumoniae *wzi*-388 allele (8). The K48 cluster was then searched by BLASTN in all the 343 ST167 genomes downloaded from EnteroBase and in the four genomes sequenced in this study, and positives were identified and highlighted in the ST167 phylogenetic tree (Fig. 2).

The K. pneumoniae K48-like capsular synthesis cluster was detected in the 100_NDM-5 and 0311_NDM-5 genomes and in another 44/343 ST167 E. coli genomes, 40 of them identified in the same branch in the NJ phylogeny tree (colored in red in Fig. 2).

In the same region in the AR_0011 (CP024855) and AR_0014 (CP024859) genomes, synteny analysis detected a complete E. coli K30-like capsular synthesis cluster (AF503612 [Fig. 3]), identified in another 8/343 genomes all belonging to the same branch of the NJ phylogeny tree (colored in pale blue in Fig. 2). In strain Y5 (CP013483), a KL115-like K. pneumoniae capsular cluster was present (data not shown), while the 91_NDM-5, 92_NDM-5, M217, and Sanji genomes showed an uncharacterized, putative capsular cluster in the same chromosomal region (Fig. 3).

As previously described, the O antigen O89b was conserved in all ST167 E. coli strains, with its gene in the locus of the *wzt* gene (positions 1339964 to 1340743 in the reference 51008369SK1 genome [CP029973]) (9), therefore distant to the locus involved in the capsular synthesis.

Another region of discontinuity within ST167 genomes was due to the presence of a type VI ICE integrated in the asparagine transfer DNA (tDNA) (Fig. 2), consisting of a type IV secretion system (T4SS), associated with the cluster encoding the yersiniabactin (Ybt) virulence trait (10, 11). The ICE element was detected in the 100_NDM-5 and 0311_NDM-5 genomes (Table 1) and in a total of 96/343 ST167 genomes downloaded from the EnteroBase database, including 7 complete sequences (Table 2). In the 51008369SK1 reference genome (CP029973), the Ybt cluster was identified at nucleotide (nt) positions 2781234 to 2810360, followed by T4SS located at nt positions 2814380 to 2825050 (Fig. 3). The 91_NDM-5 and 92_NDM-5 genomes were negative for the ICE.

A putative Ybt-like cluster showing 98% nucleotide identity with that found in 51008369SK1, but not associated with a T4SS, was identified in the AR_0150 (CP021736), AR_0151 (CP021691), AR_0149 (CP021532), and Y5 (CP013483) complete genomes (Table 2) and in 98/343 ST167 genomes from the data bank (data not shown).

## DISCUSSION

In this study, bacterial typing based on WGS was used to understand the extension of a possible interward spread of NDM-producing E. coli organisms in the hospital. Genomics demonstrated that there was not a unique, highly conserved ST167 clone in all patients. The data suggested that two pairs of strains belonged to two different variants of ST167. These variants independently entered in the hospital in different wards, probably through previously colonized patients. In one ward, coresident patients were colonized by highly related strains 91_NDM-5 and 92_NDM-5, but there were no infection cases. The other two patients in different wards were colonized or infected by strains 100_NDM-5 and 0311_NDM-5. This pair of strains showed a distinctive marker, consisting of a K48-like capsular locus shared with K. pneumoniae.

In a previously performed genomic analysis of ST167 strains, it was hypothesized that ST167 and its related ST617 lineage emerged from clone ST10 by the complete loss of the *wca* operon, encoding colanic acid biosynthesis in the lipopolysaccharide (LPS) biosynthesis pathway (12). Furthermore, a recent study proposed the O89b name for the novel variant of O89 antigen identified in ST167 detected in all strains of the clone (9). Both studies evidenced that the ST167 lineage has peculiar virulence and surface antigen features that could justify its worldwide spread as a high-risk clone. Our study brought new insights into the evolution of the ST167 clone, suggesting that this clone can acquire different capsular types. As described for other high-risk clones, such as E. coli ST131 or K. pneumoniae ST258, new clades can emerge, generating hybrid clones from the original lineage (13). In particular, the evolution of K. pneumoniae ST258 was due to a major recombination that occurred in the capsular synthesis cluster, as seems to have happened in the E. coli ST167 clone (13).

The region where the variability was observed is known to be a highly polymorphic histidine synthase-linked chromosomal region (14). In this region, the serotype-specific antigen cluster for the group 1 capsules (K1 antigens) occupies a position analogous to that of O-antigen biosynthesis genes in E. coli K-12. The altered organization in this region, relative to that in E. coli K-12, was previously hypothesized to derive from recombination events (8). The K1 antigens of E. coli were structurally related to capsules found in *Klebsiella* spp. but the capsular clusters found in ST167 were not, to the best of our knowledge, previously identified in E. coli. The presence of the K48-like cluster is consistent with the transfer of a large region of the chromosome from K. pneumoniae to E. coli or vice versa.

The global dissemination of ESBL-producing E. coli has been attributed to the rapid dispersal of a small number of E. coli lineages (12). Strains belonging to this ST167 clade K48 may globally spread and become major E. coli carbapenem-resistant high-risk clones.

## MATERIALS AND METHODS

### Ethics approval.

This study was approved by the Ethical Committee of the Policlinico Umberto I. As individual data are not being published and no intervention was performed on patients, patient consent was waived.

### Strain identification, antimicrobial susceptibility testing, and preliminary bacterial typing.

Routine identification at the species level was performed by matrix-assisted laser desorption ionization–time of flight (MALDI-TOF) mass spetrometry (Bruker Daltonics, USA) and susceptibility testing was performed by automated methods (Vitek 2; bioMérieux, Marcy L’Etoile, France). Identification of carbapenemase-encoding genes (*bla*_VIM_-, *bla*_IMP_-, *bla*_NDM_-, *bla*_OXA-48_, and *bla*_KPC_-type) was achieved by Xpert Carba-R (Cepheid, Sunnyvale, CA). The presence of the *bla*_NDM_ gene was confirmed by PCR using previously described primers (15). Strains were screened for *bla*_CTX-M-1-group_ and *bla*_CMY_ genes using primers as previously described (16, 17). NDM-producing E. coli isolates were typed by MLST according to procedures reported on the MLST website (http://mlst.warwick.ac.uk/mlst/dbs/Ecoli). Plasmid typing was performed using the PBRT 2.0 kit (Diatheva, Cartoceto, Italy).

### Conjugation and transformation.

Plasmids were extracted using the PureYield plasmid midiprep system (Promega, Italy) and transformed into E. coli K-12 MAX Efficiency DH5α chemically competent cells (Invitrogen, Italy), selecting transformants on LB agar plates containing 100 mg/liter of ampicillin. Transformants were screened by PCR for the presence of *bla*_NDM_, *bla*_CMY_, and *bla*_CTX-M-1-group_ resistance genes.

Conjugation was performed at 37°C using rifampin-resistant E. coli strain CSH26 as a recipient. Conjugants were screened by plating 10-fold serial dilutions of the mating mixture on Luria-Bertani agar solid medium plates containing 2 mg/liter of imipenem and 50 mg/liter of rifampin.

Transformation was performed using DH5α competent cells and selective plates containing 2 mg/liter of imipenem.

### Whole-genome sequencing (WGS).

Genomic DNAs were purified from 91_NDM-5, 92_NDM-5, 100_NDM-5, 0311_NDM-5, *bla*_NDM-5_-positive transconjugants, and *bla*_CTX-M-1_- and *bla*_CMY-2_-positive transformants obtained from strains 91_NDM-5 and 100_NDM-5, using the NucleoSpin tissue genomic DNA purification kit (Macherey-Nagel, Düren, Germany). DNA libraries were created using the Nextera XT DNA library preparation kit (Illumina, San Diego, CA), and sequencing was performed by the MiSeq platform 2x300PE protocol (Illumina).

*De novo* assembly of Illumina reads was performed using the SPAdes (Galaxy version 3.11.1 software at https://w3.iss.it/site/aries/). Plasmid and resistance gene content was obtained by using PlasmidFinder and ResFinder tools (https://cge.cbs.dtu.dk/services/), respectively. Replicon alleles were assigned at the plasmid MLST site (https://pubmlst.org/plasmid/). Serotype was predicted by SerotypeFinder 2.0 (https://cge.cbs.dtu.dk/services/). Genome synteny was performed by SEED Viewer version 2.0 (http://rast.nmpdr.org/seedviewer.cgi). Genome sequences were annotated at the RAST server (http://rast.nmpdr.org/).

### Nanopore sequencing and plasmid assembly.

Nanopore sequencing was performed on DNA isolated from strains 91_NDM-5 and 100_NDM-5 using the NucleoSpin tissue genomic DNA purification kit (Macherey-Nagel, Düren, Germany). About 400 ng of DNA was used for library preparation. The rapid DNA ligation kit (SQK-RBK004) from Nanopore was utilized to prepare the library, and the library was sequenced using R9.4.1 chemistry. Bacterial genomes and plasmids were assembled with Mini_assembler software from the package Pomoxis (https://github.com/nanoporetech/pomoxis).

Complete plasmid sequences were obtained combining the Nanopore and Illumina assembly. Plasmid sequences were annotated at the RAST server (http://rast.nmpdr.org/) and manually curated in Sequin Application version 16.0 annotation software.

### Phylogenetic analysis.

Cluster analysis of ST167 genomic sequences was performed by building a neighbor-joining (NJ) tree on an SNP analysis performed by the kSNP version 3.0 software at the ARIES public Galaxy server (https://w3.iss.it/site/aries/). For comparison, 343 ST167 genomes were downloaded from the Enterobase database (http://enterobase.warwick.ac.uk/species/index/ecoli) and included in the analysis (data not shown).

The phylogenetic tree was visualized using the Fig Tree program version 1.3.1 (http://tree.bio.ed.ac.uk/software/figtree/).

### Data availability.

Genome and plasmid sequences have been deposited in GenBank (https://www.ncbi.nlm.nih.gov/pubmed) under BioProject no. PRJNA545093. Strains were stored under BioSample numbers as follows: 91_NDM-5, SAMN11872784; 92_NDM-5, SAMN11872786; 100_NDM-5, SAMN11872785; and 0311_NDM-5, SAMN11872787. Manually curated plasmid sequences were released under GenBank accession numbers as follows: p91_CMY-42, MN007140; p91_NDM-5, MN007141; p100_CTX-M-1, MN007142; p100_NDM-5, MN007143; and p100_NDM-5_CTX-M-1, MT199177.
