# Supplementary material for: Novel Insights and Features of the NDM-5-Producing Escherichia coli Sequence Type 167 High-Risk Clone
Source: mSphere. 2020 Apr 29;5(2):e00269-20. doi: 10.1128/mSphere.00269-20 (PMC7193042; doi:10.1128/mSphere.00269-20)

Supplementary Figure 1

Reference	51083696SKI O89b:K48:H9
1	100_NDM-5 O89b:K48:H9
2	91_NDM-5 O89b:H9
3	AR_0011 O89b:O9:H9
4	AR_0014 O89b:H9
5	CREC-629 O89b:H10
6	ECONIH6 O89b:K48:H9
7	FDARGOS434 O89b:K48:H9
8	M217 O89b:H9
9	Sanji O89b:H9
10	SCEC020007 O89b:K48:H9

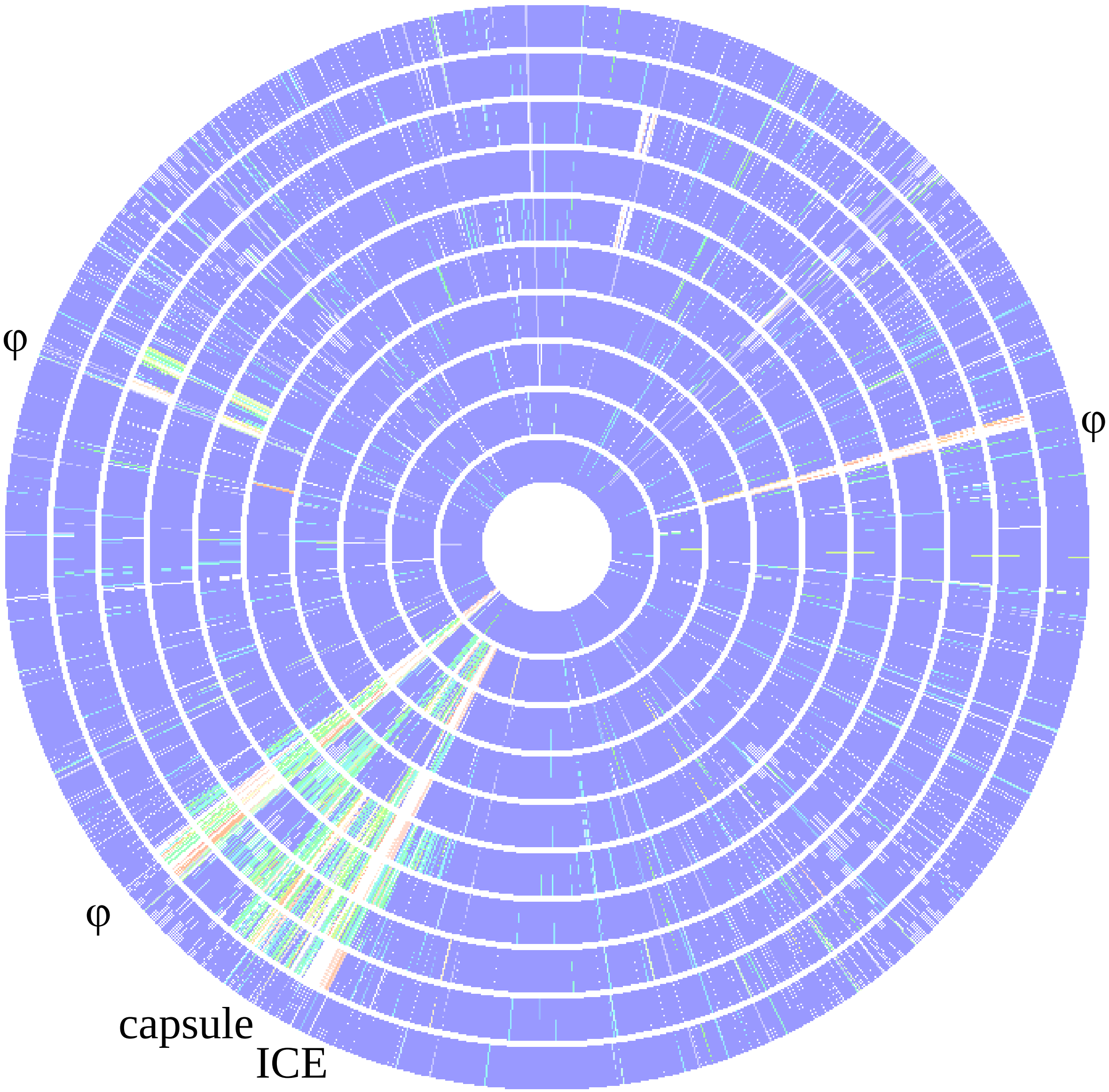

Supplement: FIG S1 [file mSphere.00269-20-sf001.pdf]
